# Simultaneous fusion, imaging and encryption of multiple objects using a single-pixel detector

**DOI:** 10.1038/s41598-017-12664-1

**Published:** 2017-10-13

**Authors:** Shi Dongfeng, Huang Jian, Wang Yingjian, Yuan Kee, Xie Chenbo, Liu Dong, Zhu Wenyue

**Affiliations:** 1 Key Laboratory of Atmospheric Optics, Anhui Institute of Optics and Fine Mechanics, Chinese Academy of Sciences, Hefei, 230031 China; 2 University of Science and Technology of China, Hefei, 230026 China

## Abstract

A novel technique for the simultaneous fusion, imaging and encryption of multiple objects using a single-pixel detector is proposed. Here, encoded multiplexing patterns are employed to illuminate multiple objects simultaneously. The mixed light reflected from the objects is detected by a single-pixel detector. An iterative reconstruction method is used to restore the fused image by summing the multiplexed patterns and detected intensities. Next, clear images of the objects are recovered by decoding the fused image. We experimentally obtain fused and multiple clear images by utilizing a single-pixel detector to collect the direct and indirect reflected light. Technically, by utilizing the patterns with per-pixel exposure control, multiple objects’ information is multiplexed into the detected intensities and then demultiplexed computationally under the single-pixel imaging and compressed sensing schemes. An encryption experiment is performed by setting the multiplexed patterns’ encoding as keys.

## Introduction

With the development of semiconductor technology, traditional imaging matrix detectors (such as charge-coupled devices [CCDs] and complementary metal-oxide-semiconductors [CMOSs]) tend to have large arrays of small pixels. The latest matrix detectors can capture high-resolution images with billions of pixels. Alternatively, an imaging system^1–7^ based on an un-scanned single-pixel detector (such as a photodetector [PD] or photomultiplier tube [PMT]) has received increasing attention from researchers in recent years. This single-pixel imaging (SPI) system employs random patterns to illuminate the object, and then the intensities of the reflected or transmitted light from the object are acquired by an un-scanned single-pixel detector. Subsequently, the image of the object can be recovered based on the correlation between the illumination patterns and the detected intensities. This new imaging system can reduce the cost or size of matrix detectors, especially in the infrared and terahertz region of the spectrum, where matrix detectors do not have such good specifications compared to their performance in the visual spectrum. Recently, SPI systems for image encryption^8–13^ have also been studied. Marked method^12^ has been proposed to encryption. An optical cryptosystem^13^ based on SPI system has also been presented by using single-pixel encoding and the Gerchberg–Saxton algorithm. In general, the random patterns or/and the detected intensities are always utilized as keys in SPI encryption systems. To best of our knowledge, there is no a SPI system can be simultaneously achieve multi-object fusion, imaging and encryption. Here, an SPI technique comprising multi-object fusion, imaging and encryption is proposed, and an experimental study to validate this SPI technique is conducted.

This technique originated from compressed sensing (CS) theory^14^ that allows the recording an image consisting of *N* ^*2*^ pixels using much fewer than *N* ^*2*^ measurements if it can be transformed to a basis where most pixels have negligibly small values. For example, theoretically, a 1-mega-pixel array could potentially be used to reconstruct a 4-mega-pixel image. Specifically, a random sample of the 4-mega-pixel image is taken and then recovered through sparse signal reconstruction methods. This is possible because natural images tend to be sparse (i.e., only a small fraction of these projections have relevant information) in some bases of functions. The hardware limitations of traditional imaging systems in terms of their spatial resolution and temporal resolution can be effectively addressed using this characteristic. To date, several matrix imaging systems^15–17^ have been built, and the effectiveness of the theory has been confirmed experimentally. For example, a method of efficient space-time sampling with pixel-wise coded exposure to reconstruct a video from a single coded image while maintaining high spatial resolution has been proposed^15^. Additionally, Liang *et al*. have accomplished single-shot compressed ultrafast photography at one hundred billion frames per second with random sampling^16^. Collecting the spectral information of an imaging scene via random sampling has also been proposed^17^. Simultaneously, the CS algorithm has also received substantial attention for SPI. Nevertheless, in the above SPI-related refs^1–7^, the imaging scene is completely sampled by the illumination patterns. However, according to the CS analysis, accurate image information can be recovered when the scene is incompletely sampled by illumination patterns. Then, patterns with observed in spatial multiplexing mode can be used to simultaneously sample multiple objects in an orderly manner. In other words, an SPI system with spatial multiplexed patterns can be employed to achieve the fusion, imaging and encryption of multiple objects.

**Figure 1:**
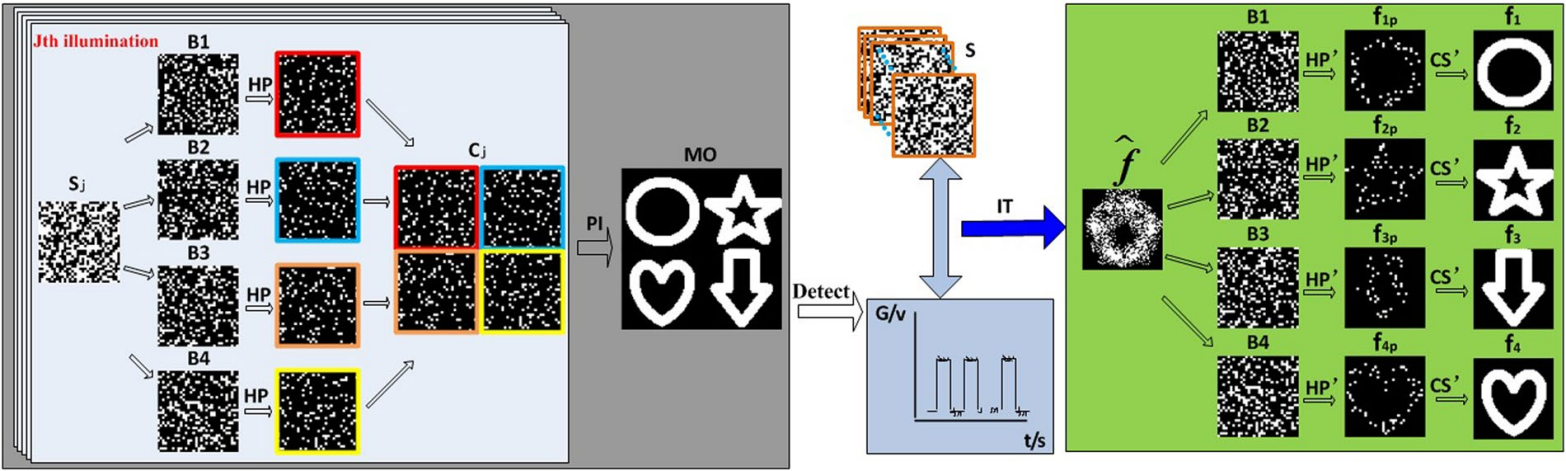
Procedure of the proposed method: S:the array of multiplexed patterns, S_j_: the j-th multiplexed pattern; B1–B4: four complementary binary encoded matrices; C_j_: the j-th illumination pattern; HP: Hadamard product of S_j_ and the encoded matrices; PI: projection illumination; MO: multiple objects; G: intensities detected by the single-pixel detector; IT: iterative reconstruction method that is used to recovered fused image; HP’: Hadamard product of the fused image $\widehat{f}$ and the encoded matrices; f_1p_, f_2p_, f_3p_ and f_4p_: four random sampling images_;_ CS’: CS algorithm that is used to compute images of four objects; f_1_, f_2_, f_3_ and f_4_: four finally-recovered images. Single arrows describe process, and double arrows describe interaction of the parameters at both ends of the arrow.

The whole procedure of the proposed technique is demonstrated in Fig. 1, in which four objects serve as an example. First, the computer program produces four complementary binary encoded matrices—B1, B2, B3, and B4—and the array of multiplexed patterns S. The illumination patterns C are produced by resizing the matrices obtained by multiplying the multiplexed patterns and encoded matrices. The assembling procedure of illumination pattern C_*j*_ is shown in the gray frame of Fig. 1. The illumination patterns are loaded into the projection system and then projected onto the scene. Each subpart of the illumination patterns can illuminate the corresponding object without crossing. The reflected light intensity G of the four objects is detected by a single-pixel detector. The fused image of the four objects can be restored using an iterative reconstruction method. Next, four random sampling images named f_1p_, f_2p_, f_3p_ and f_4p_, indicating different random partial object information, can be recovered by multiplying the fused image and encoded matrices. Finally, the original images of the corresponding object can be obtained using the CS algorithm. Here, multiple images are fused and encrypted by integrating them in the mixed measurement. When the encoded matrices are unknown, accurate object information cannot be obtained. Accordingly, using the encoded matrices of the proposed method as keys can result in the encryption of the objects’ information. The green part in the figure is multi-object demultiplexing process.

## Results

### Experimental setup

The experimental system used to study the proposed approach is described as follows. A 532-nm continuous wave (cw) laser serves as the light source. A digital micro-mirror device (DMD: TI DLP 6500 system) is used to generate illumination patterns. Single-pixel detectors (Thorlabs PMT-PMM02) and data acquisition system (NI USB-6211) are employed for light detection and data acquisition, respectively. The laser beam enters a beam expander (BE) and then passes through the DMD, which provides time-varying illumination patterns. The direct and indirect reflected light from the multiple objects (MO) is collected by collecting lens and measured by the single-pixel detector, and then the intensity data are sent to a computer through the data acquisition system (DAS). The DMD system with per-pixel exposure control can achieve the anticipant illumination patterns, which is the foundation of the successful implementation of the proposed technique.

Various multiplexed patterns can be employed in the SPI system. Random patterns, in which each mask has a random distribution of binary values, have been widely used. In this case, complex operations are required to obtain the object information. Hadamard patterns provide another strategy that enables the reconstruction of the image with a linearly iterative algorithm, which requires very low computational complexity. Thus, Hadamard multiplexed patterns are chosen for our experiments. The basis of Hadamard patterns is composed of orthogonal discrete square waves, with values of either +1 or −1. Because the illumination patterns produced by the DMD system are binary, positive and negative reflection values cannot be readily utilized. To address this issue, two approaches can be employed. The first approach involves the use of transformative Hadamard matrices and ensures that all entries consist of values of either 1 or 0. The second approach involves a pair of matrices that are related to the Hadamard matrix by a subtraction operation. The second approach can improve the signal-to-noise ratio (SNR) of the measurements^7^ and is utilized in our system. The process is described as follows. The entries of a Hadamard matrix H are either +1 or −1. We create the complementary pair H_±_ = (E ± H)/2, where E represents a matrix for which all entries are equal to 1. As a result, we have one matrix H_+_ where the original +1 entries retain their value, and the −1 entries become zero. In the other matrix H_−_, unity-valued entries become zero, and the −1 entries become 1. As the detected intensity is linear with the patterns, the patterns with H_+_ and H_−_ patterns are employed in the projection; thus, the coefficient under the Hadamard basis can be calculated by subtracting the two detected intensities.

**Figure 2:**
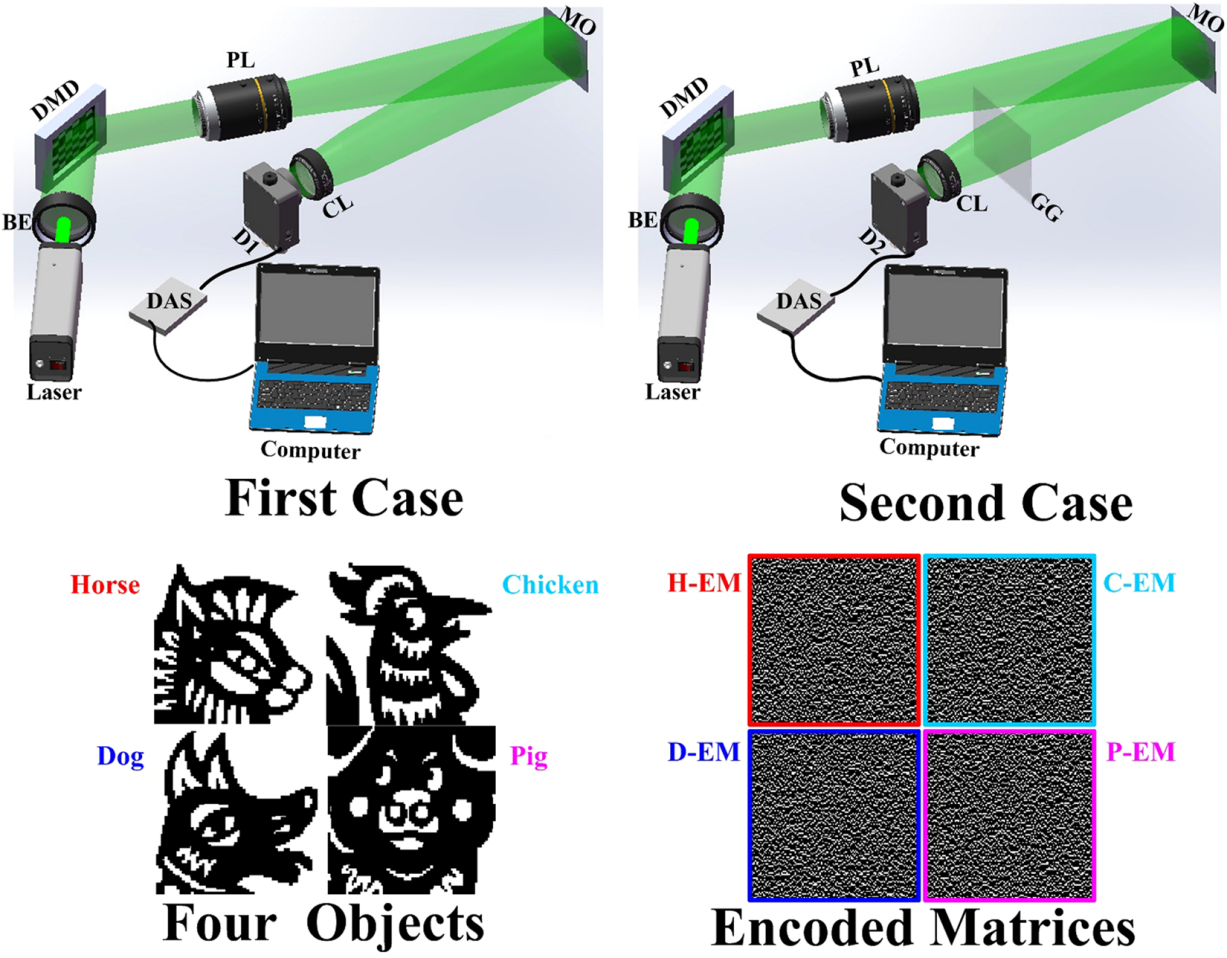
The configuration of the SPI system for the simultaneous fusion, imaging and encryption of multiple objects. PL: projection lens with 100 mm focal length; MO: multiple objects; CL: collecting lens with 75 mm focal length; GG: Ground Glass; D1 and D2: single-pixel detectors. H-EM, C-EM, D-EM and P-EM labeled with different color borders are encoded matrices applied to four cartoon binary animals (horse, chicken, dog and pig), respectively.

Four binary cartoon animals (horse, chicken, dog and pig) printed on A4 paper are employed as the multiple objects in the experiment and are placed side by side at the object position, as shown in Fig. 2. The objects are located at a distance of **~**1.9 m from the imaging system. The 3 × 3 mirrors of the DMD are combined into a pattern cell that corresponds to an image pixel, and the intermediate 768 × 768 mirrors are utilized in the experiment. Thus, the resolution of the entire lighted area is 256 × 256 pixels. The imaging area is divided into four areas; thus, the imaging resolution is 128 × 128 pixels. Four 128 × 128 pixel encoded matrices labeled with borders of different colors are shown in Fig. 2 and arranged in a 2 × 2 manner. These matrices were produced by a random process and have complementary orthogonal properties with a compression ratio of 25%. During the acquisition process, the encoded matrices remain unchanged. The pre-process is described as follows. First, 16384 pairs of complementary Hadamard patterns are generated as multiplexed patterns with values of 0 or 1 according to the above method. Next, four-combined illumination patterns with 768 × 768 mirrors are reproduced by arranging and resizing intermediate matrices via multiplying the Hadamard patterns with the encoded matrices and are then loaded into the DMD system. Finally, the four-combined illumination patterns are employed to illuminate the four objects, and the direct and indirect reflected lights from four objects is collected by the single-pixel detectors. Here, each subpart of the illumination patterns can illuminate the corresponding object without crossing. We carry out three different experiments. In the first case, the direct reflected light from the four objects is collected by the collecting lens and detected by detector 1. In the second case, the reflected light from the four objects passes through ground glass, and then, the indirect reflected light is detected by detector 2. In the third case, the reflected light from the four objects is reflected by a sheet of white A4 paper, and then, the indirect reflected light is detected by detector 3, which is a reverse-oriented photodiode without a direct view of the objects. The fusion, imaging and encryption of the multiple objects are achieved using the intensities detected by the detectors, respectively. The configuration and experimental results of the third case are shown in Supplementary Material.

### Experimental results of multi-object fusion and imaging

**Figure 3:**
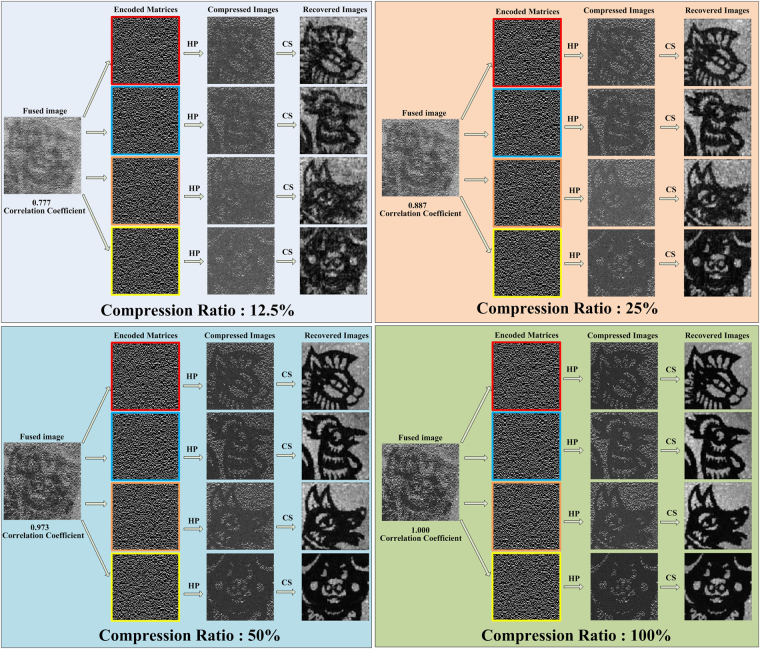
Multiple objects reconstructed with different compression ratios using direct reflected light in the first case. Scene reconstructed with different compression ratios according to the correlation coefficient between the recovered images and the reconstruction utilizing a complete Hadamard basis (100% compression ratio).

An evolutionary linear iterative method^7^ aiming to reconstruct the image in significantly less time than conventional CS is employed to recover the fused image. The evolutionary linear iteration scheme chooses a subset of the Hadamard patterns to recover the fused image of the multiple objects by selecting the patterns with the most significant intensities measured by the single-pixel detector. In the first case, the fused image recovered from different compression ratios are shown in Fig. 3. Based on the results, the SNRs of the fused images increase as the compression ratio increase. However, we cannot identify the multi-object information from fused images. Fortunately, based on the properties of encoded matrices, four compressed images can be obtained by multiplying the fused image with the encoded matrices. At last, the results of the finally recovered images are obtained using CS algorithm. The experiment was determined to be successful based on the fact that completely accurate image information can be recovered exactly from compressed samples. The results show that the quality of recovered images is affected by the SNR of the fused image and that a positive correlation exists between them. The differences among the images reconstructed from the fused images with coverage spanning compression ratios from 50 % to 100% are nearly negligible. The results are shown in Fig. 3, and each partition represents a multi-object demultiplexing process at different compression ratios. Note that the images are not subjected to any additional processing, such as filtering.

**Figure 4:**
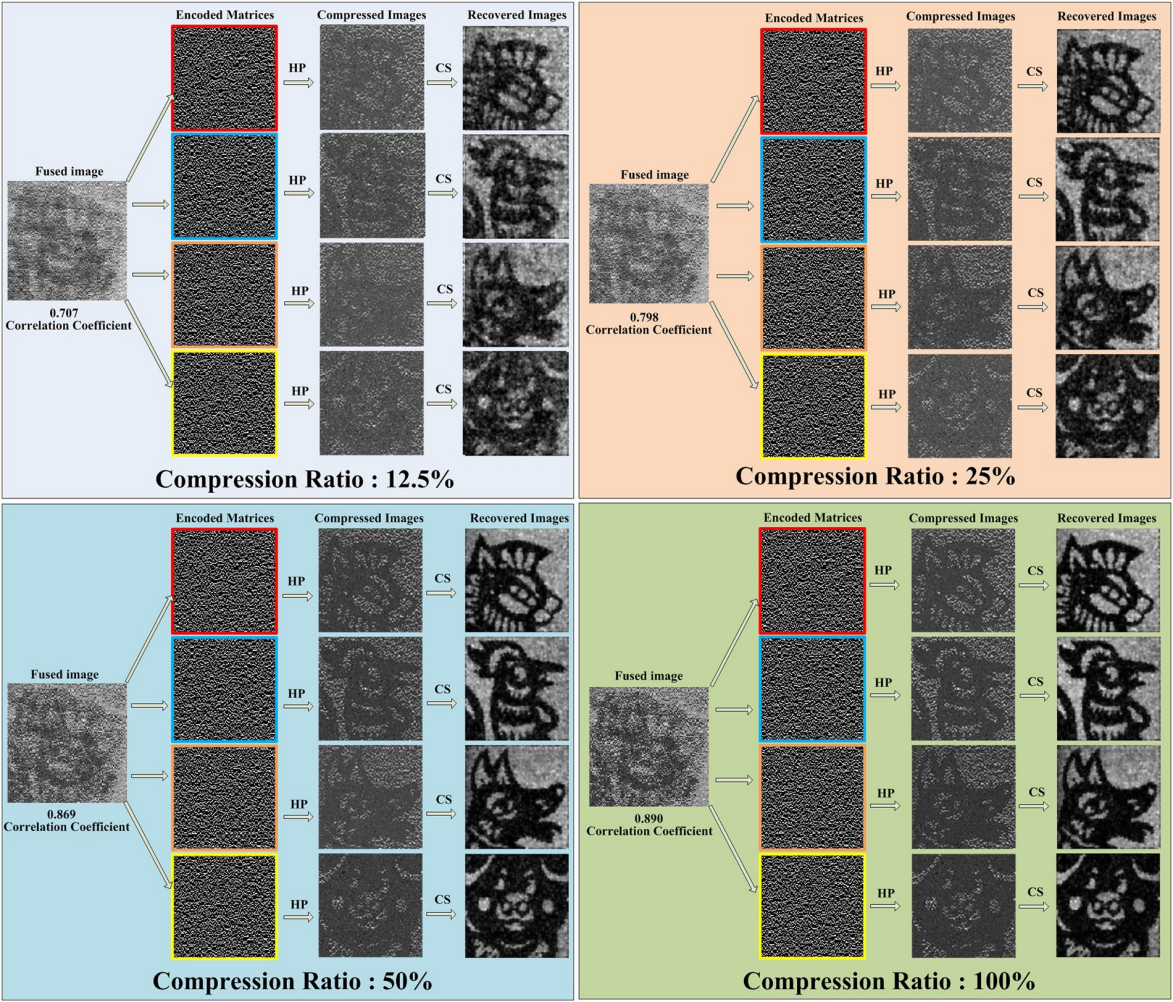
Multiple objects reconstructed with different compression ratios using indirect reflected light in the second case. Scene reconstructed with different compression ratios according to the correlation coefficient between the recovered images and the reconstruction utilizing a complete Hadamard basis (100% compression ratio).

Scattering media limit optical observations because their random refractive index variations distort the spherical wavefront generated by every point source, resulting in a smeared image at a matrix detector^18^. The following two experiments are employed to show that the proposed method can also work in scattering environment. In the second case, the reflected light from the objects passes through the ground glass before being collected by the collecting lens and directed into detector. The results are shown in Fig. 4. These images appear noisy because the light collected by detector was relatively weak as a result of the low transmittance of the ground glass, resulting in a detection signal with a lower SNR. Based on the correlation coefficient with the reconstruction utilizing complete Hadamard basis for direct reflected light, the fused result improves as the number of multiplexed patterns increases, and the best result is similar to that obtained with a compression ratio of 25% in the above situation. The results indicate that we can clearly identify multi-object information in scattering environment. The ability to image multiple objects through scattering media makes our system valuable in numerous applications, ranging from optical communication through turbulent atmosphere to microscopic imaging in turbid tissues. The experimental results of the third case are shown in Supplementary Material.

### Experimental encryption results

**Figure 5:**
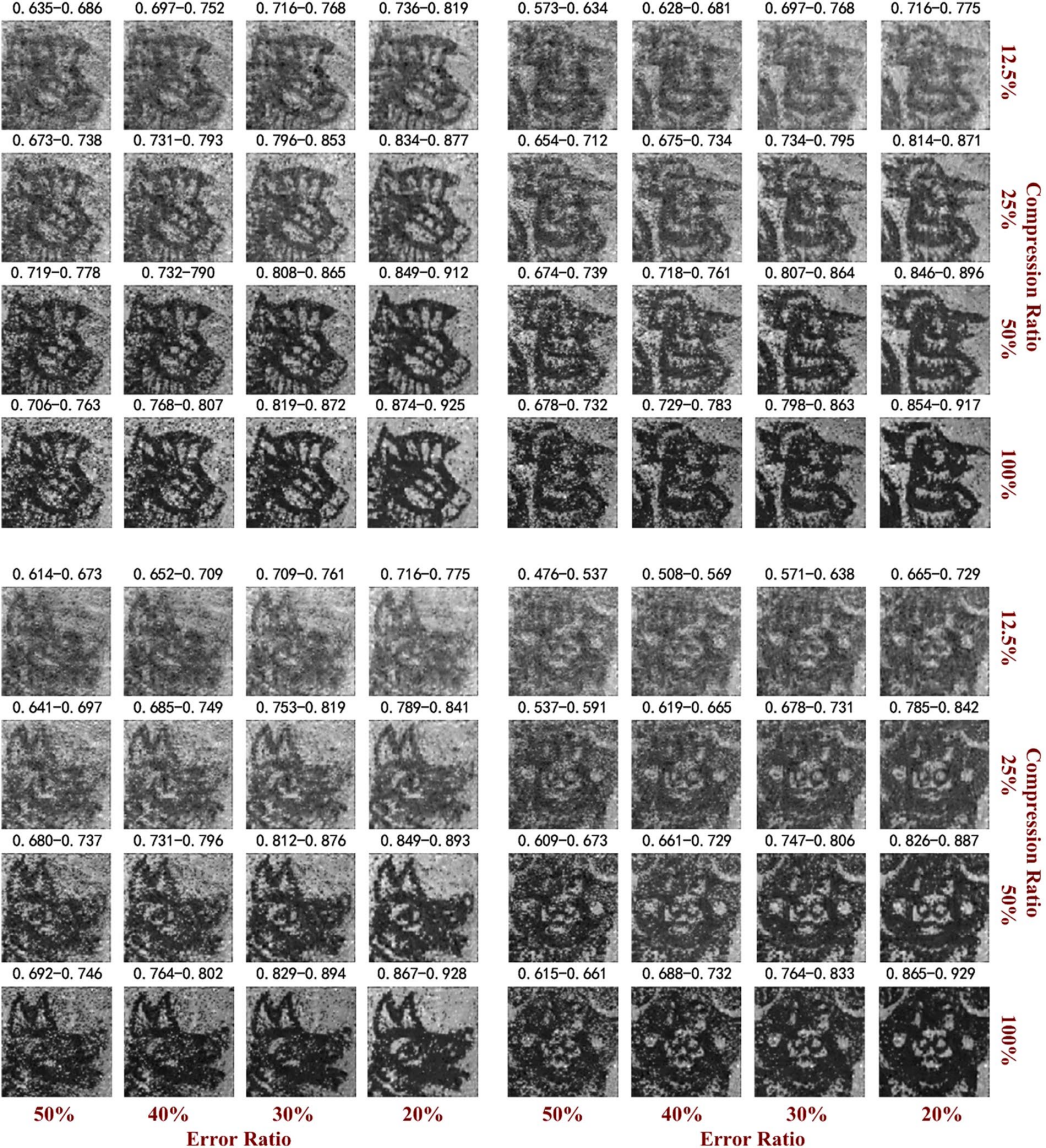
Image encryption experiment using direct reflected light in the first case. The results obtained under error ratios of 50%, 40%, 30% and 20% for the encoded matrices are shown. The numbers indicate the correlation coefficients between the recovered images and the reconstruction utilizing the complete Hadamard basis for direct reflected light (100% compression ratio).

**Figure 6:**
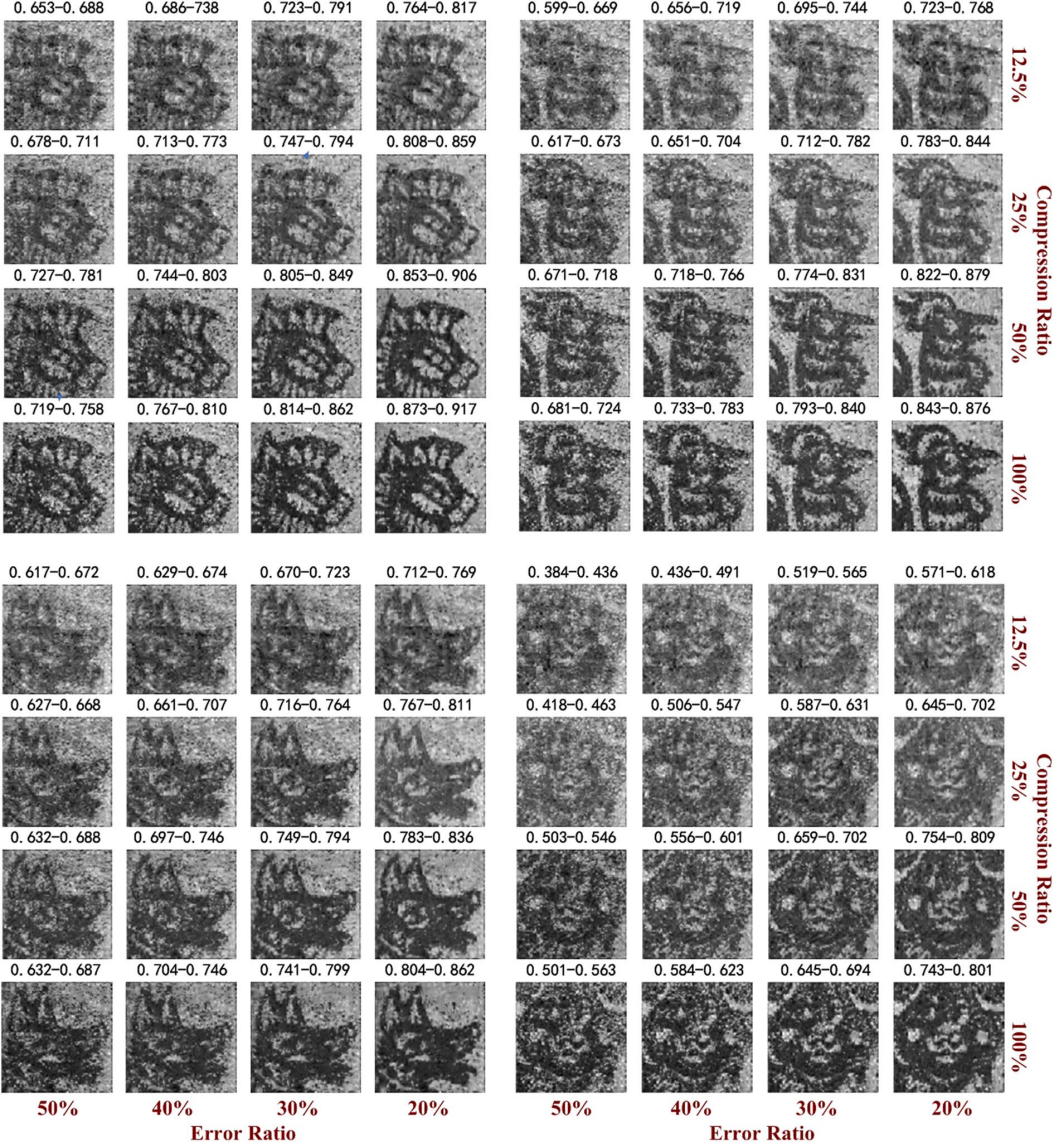
Image encryption experiment using indirect reflected light in the second case. The results under error ratios of 50%, 40%, 30% and 20% for the encoded matrices are shown. The numbers indicate the correlation coefficients between the recovered images and the reconstruction utilizing the complete Hadamard basis for direct reflected light (100% compression ratio).

In prevenient SPI encryption systems, the random patterns or/and the detected intensities are always utilized as keys. Here, we use encoded matrices as key and the experimental results to verify the system’s encryption capability. In the information encryption transmission process, the detected intensities and multiplexed patterns are transmitted as public information, and only the four encoded matrices are transmitted in an encrypted manner. It is assumed that, during the information transmission, the eavesdropper obtains multiplexed patterns and intensities detected by the single-pixel detector, determines the encoded matrices with a certain level of accuracy, and then uses the partial accurate information to restore the images of the four encrypted objects. For direct comparison, the reconstructed results of the four objects are produced by the encoded matrices with a certain level of accuracy and shown in Fig. 5. According to the results, as the bit error ratios decrease, the quality of the recovered images gradually improves. The quality of the recovered images also improves as the compression ratio increases. We imitate a brute force attack. Fifty encoded matrices with a certain level of accuracy and different distributions are generated as decryption keys. In each attack, the reconstruction using encoded matrices with a certain level of accuracy retrieve the information. The minimum and maximum values of the correlation coefficients in fifty different cases are shown in Fig. 5. The correlation coefficients in the figure demonstrate that our system is resistant to brute force attacks. Note that the restored image is simultaneously affected by the bit error ratio and the number of multiplexed patterns. To obtain high-quality object information, an eavesdropper not only requires high-precision encoded matrices but must also use more patterns to restore the image. Image encryption in a scattering environment was also performed. Figure 6 shows the restoration of multiple objects at different bit error ratios and different numbers of Hadamard basis using the indirect reflected intensities from detector 2. The results show that the relationship between the restored image quality and the error ratio is the same as that determined for the above situation. The system’s encryption performance can be further improved by the encrypted transmission of patterns or/and intensities. For the sake of brevity, performance of other security keys, such as patterns or/and intensities or some combination of the three parameters, is not presented here.

Advantages of the proposed method and its comparisons to previous works are discussed as follows:

First, multiple objects can be fused, imaged and encrypted using sub-Nyquist measurements, which significantly reduces the data acquisition and allows faster data communication to users. Therefore, the system efficiency can be greatly increased using the proposed method.

Second, the united process, which incorporates the encryption of the fused image with the compressed acquisition of multiple objects, ensures the security of private content and, thus improves the encryption performance of the SPI system relative to conventional single-pixel encryption methods (in which patterns or/and detected intensities are employed as keys) by providing a third key based on the encoded matrices used in this method.

Third, in the traditional SPI system for imaging, each object must be individually measured once to maintain the same image resolution. In order to recover an image of object, SPI system needs to project a set of illumination patterns produced by DMD. The size of this set depends on the desired resolution of image. Even for low resolution image, it requires a huge amount of illumination patterns. In spite of the fact that DMD which has very high modulation speed is usually used in SPI system, this also limits the speed of the acquisition process compared with conventional techniques. The maximum frame rate of reconstructed image with 64 × 64 pixel resolution is about 10 Hz when the DMD displayed at a rate of 20.7 kHz^6^. In this situation, if we use a sequential approach to image four objects, the required exposure time is 0.4 s. However, the required exposure time is 0.1 s, when the proposed method is employed. The advantage of proposed method will be more pronounced when more objects are imaged. Thus, the proposed method can effectively improve the imaging efficiency.

Forth, in conventional optical systems for achieving multi-object fusion, imaging and encryption, matrix detectors are usually applied^19,20^. In this study, a single-pixel detector is employed to achieve the same objective; hence there is a potential to establish lower-cost and more compact systems, especially in the infrared and terahertz region of the spectrum, where matrix detectors do not have such good specifications compared to their performance in the visual spectrum. Moreover, in weak light environment, SPI system can obtain image with higher SNR than that recorded by matrix detectors^5^.

## Discussion

Based on multiplexed patterns, an SPI technique for multi-object fusion, imaging and encryption is developed and tested experimentally. The results revealed that the proposed method enables the simultaneous imaging of multiple objects, even in a scattering environment, and is highly efficient for information encryption. Note that in this paper, the fused image contains four objects’ information; thus, the compression ratio is a quarter of the above value relative to the whole information. In other words, the proposed method realizes the fusion, imaging and encryption of multiple objects via sub-Nyquist measurements. The fusion, imaging and encryption of more objects can be achieved by further compressing the encoded matrices.

In general, the central challenge addressed by this method is to find an architecture that effectively balances the final recovery image quality with the number and pattern of the encoded matrices. The choice of the encoded matrix plays a key role in image reconstruction. Because various matrices can be chosen, the problem of optimizing the encoded matrix should be carefully studied in the future. However, when we use this method to encrypt multiple objects, the optimal encoded matrix with high encryption performance and transmission efficiency can be pre-selected by investigating the sparse characteristics of the transmitted information. The number and resolution of recovered images are limited by the number of pixels of the device (DMD, SLM). However, this problem can be mitigated by splicing multiple devices (DMD, SLM). In addition, due to environmental interference, light source instability and other factors, the quality of the information acquired by the SPI system is not high; thus, efficient system configuration and restoration algorithms must be explored. We believe that this new technique will pave the way for the use of this SPI system in several fields, including optical communication, imaging around corners, and especially imaging multiple objects in a scattering medium, such as water or fog.

## Method

The reconstruction of the proposed technique involves two main steps: spatial multiplexing and multi-image demultiplexing.

### Spatial Multiplexing

When we have multiple objects *f*_1_, *f*_2_…… and *f*_*i*_ to fuse, image and encrypt via SPI, different encoded matrices *B*_1_, *B*_2_…… and *B*_*i*_ with resolutions of *N × N* are employed to sample the multiple objects. It is assumed that the *jth* illumination pattern *C*_*j*_ is generated by arranging the matrices achieved by multiplying multiplexed pattern *S*_*j*_ and *B*_1_, *B*_2_……*B*_*i*_ and can be expressed as:

1 $$\begin{array}{c}{C}_{j}(1\,:\,N,1\,:\,N)={B}_{1}\circ {S}_{j},\\ {C}_{j}(1\,:\,N,N+1\,:\,2N)={B}_{2}\circ {S}_{j},\\ \ldots \\ {C}_{j}(1\,:\,N,(i-1)N+1\,:\,iN)={B}_{i}\circ {S}_{j},\end{array}$$

Each sub-region with *N* × *N* pixels of illumination patterns corresponds to an object; thus, each illumination pattern can illuminate several objects simultaneously. The resolution of the restored image is equal to 128 × 128. The sub-regions of the above formula are arranged in columns for simplicity. In the actual system, the sub-regions can be arranged as required. In the system described here, four sub-regions are arranged in a 2 × 2 manner. The matrices of the illumination patterns are loaded into the DMD system via a computer. Multiple objects are placed in each division of the illumination pattern, and then, a series of illumination patterns are employed to illuminate multiple objects simultaneously.

The reflected intensities from the multiple objects can be expressed as

2 $$\begin{array}{c}{g}_{j,1}={\sum }_{N\times N}{B}_{1}\circ {S}_{j}\circ {f}_{1},\\ {g}_{j,2}={\sum }_{N\times N}{B}_{2}\circ {S}_{j}\circ {f}_{2},\\ \mathrm{...}\\ {g}_{j,i}={\sum }_{N\times N}{B}_{i}\circ {S}_{j}\circ {f}_{i},\end{array}$$

The whole reflection intensity from several objects is detected by a single-pixel detector. Thus, the detected intensities of the single pixel detector can be written as

3 $$\sum _{i}{g}_{j,i}={\sum }_{N\times N}{B}_{1}\circ {S}_{j}\circ {f}_{1}+{\sum }_{N\times N}{B}_{2}\circ {S}_{j}\circ {f}_{2}+\mathrm{...}+{\sum }_{N\times N}{B}_{i}\circ {S}_{j}\circ {f}_{i},$$

Suppose binary encoded matrices have the following properties:

4 $${B}_{i1}\circ {B}_{i2}=\{\begin{array}{cc}0 & i1\ne i2\\ {B}_{i1} & i1=i2\mbox{'}\end{array}$$

5 $$\prod {B}_{i}=E,$$

where *E* represents a matrix with all entries are equal to 1, and $\prod$ indicates the accumulation of all matrices. In other words, multiple encoded matrices achieve complete sampling of the fused object information. In addition, they are orthogonal to each other. Further simplifying Eq. (3), we have

6 $${g}_{j}={\sum }_{N\times N}{S}_{j}\circ \hat{f},$$

where fused image $\hat{f}={B}_{1}\circ {f}_{1}+{B}_{2}\circ {f}_{2}+\mathrm{...}+{B}_{i}\circ {f}_{i}$ represents the accumulation of the random samples from multiple objects, and ${g}_{j}=\sum _{i}{g}_{j,i}$. From Eq. (6), we can determine that the acquisition process can be described by the interaction between the multiplexed patterns and the fused image. Multiplexed Hadamard patterns are used in this paper. As the illumination patterns produced by the DMD system are binary, we create complementary pairs of illumination patterns H_+_ and H_-_. The intensities detected when illumination patterns H_j+_ and H_j-_ are used to illuminate the objects can be expressed as g_j+_ and g_j-_, respectively, and then, the two intensities are subtracted. This process can be written as

7 $${g}_{j}={g}_{j+}-{g}_{j-}={\sum }_{N\times N}({H}_{j+}\circ \hat{f}-{H}_{j-}\circ \hat{f})={\sum }_{N\times N}({S}_{j}\circ \hat{f}),$$

The iterative reconstruction method is employed to recover the fused object information, which can be expressed as

8 $$\hat{f}={S}_{1}\circ {g}_{1}+{S}_{2}\circ {g}_{2}+\mathrm{...}+{S}_{j}\circ {g}_{j}=\sum _{j}{S}_{j}\circ {g}_{j},$$

where $\hat{f}$ is the recovered fusion image. Here, the number of the multiplexed patterns is equal to *j*. The above formula indicates that the fused image can be expressed as a weighted sum of multiplexed patterns based on the corresponding coefficients obtained from the detected intensities. The compression ratio is defined as the ratio of the number of multiplexed patterns to image pixels:

9 $$cr=j/{N}^{2}\times 100 \% .$$

During this analysis, a pattern cell is utilized as an image pixel.

### Multi-object Demultiplexing

The above equation demonstrates that when the reflected intensities from multiple objects are detected, they are mixed together. Thus, the image of multiple objects is recovered by iterative reconstruction method. However, we must obtain the image of each individual object. Fortunately, based on the properties of encoded matrices, random samples of each object can be obtained as follows:

10 $${\hat{f}}_{i}={B}_{i}\circ \hat{f}={B}_{i}\circ ({B}_{1}\circ {f}_{1}+{B}_{2}\circ {f}_{2}+\mathrm{...}+{B}_{i}\circ {f}_{i})={B}_{i}\circ {f}_{i},$$

From the above formula, the Hadamard product of the fused image and encoded matrices can be used to obtain the corresponding random object information. Next, the complete information of each object can be determined with high precision by substituting the encoded matrices and the random sampled image in the CS algorithm. Optimizations can be achieved as follows:

11 $${f}_{i}=y{a}_{i}\,{\rm{subjecttomin}}\{{\Vert {\hat{f}}_{i}-{B}_{i}y{a}_{i}\Vert }_{2}^{2}+\lambda T({a}_{i})\},$$

Here, *y* represents the transform to the chosen domain resulting in a sparse representation *a*_*i*_, λ and *T* represent the regularization coefficient and function, respectively. The code employed for CS in this paper^21^ is the function *Inpainting_GSR* of the software package. According to the above analysis, when the accurate encoded matrix cannot be obtained, accurate images of the objects cannot be recovered; that is, the encoded matrix can be utilized as a key to encrypt the object.

## Electronic supplementary material

Supplementary Material
