# Supplementary material for: Simultaneous fusion, imaging and encryption of multiple objects using a single-pixel detector
Source: Sci Rep. 2017 Oct 13;7:13172. doi: 10.1038/s41598-017-12664-1 (PMC5640669; doi:10.1038/s41598-017-12664-1)
Supplement: Supplementary file 1 — Supplementary Material [file 41598_2017_12664_MOESM1_ESM.doc]

**Supplementary Material**

Simultaneous fusion, imaging and encryption of multiple objects using a single-pixel detector

Shi Dongfeng^1a)^, Huang Jian^1,2*^, Wang Yingjian^1,2^, Yuan Kee^1^, Xie Chenbo^1^, Liu Dong^1^ and Zhu Wenyue^1^

^1^Key Laboratory of Atmospheric Optics, Anhui Institute of Optics and Fine Mechanics, Chinese Academy of Sciences, Hefei 230031, China

^2^University of Science and Technology of China, Hefei 230026, China

[^A^Email](mailto:*opex@osa.org；AEmaile): dfshi@aiofm.ac.cn;*Email: jhuang@aiofm.ac.cn


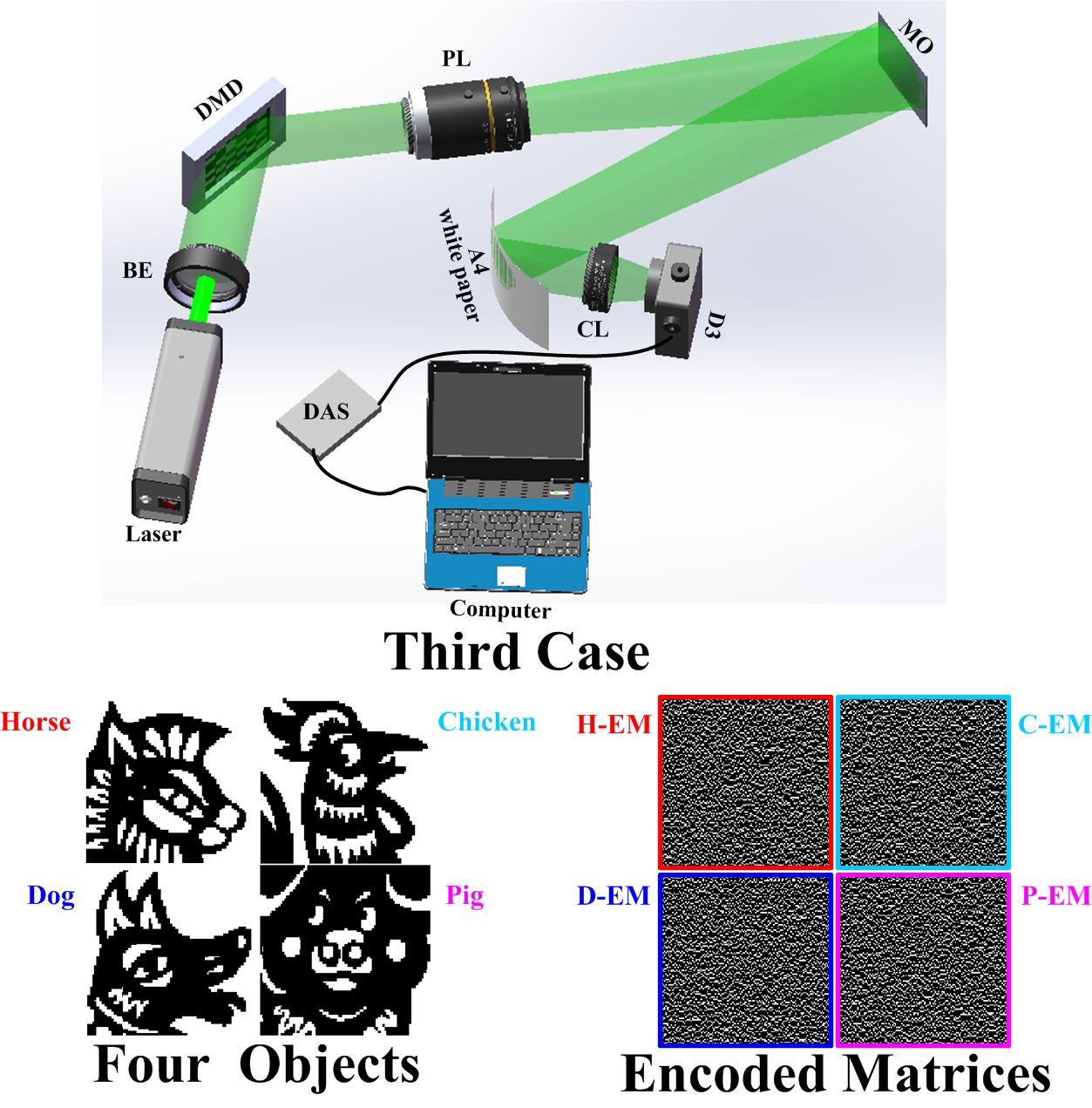


Fig. 1. The configuration of the SPI system for the simultaneous fusion, imaging and encryption of multiple objects in a scattering environment. BE: beam expander; DMD: digital micro-mirror device; PL: projection lens; MO: multiple objects; CL: collecting lens; DAS: data acquisition system; D3: single-pixel detector. H-EM, C-EM, D-EM and P-EM labeled with different color borders are encoded matrices applied to four cartoon binary animals (horse, chicken, dog and pig), respectively.

The reflected light from the four objects is reflected by a sheet of white A4 paper, and then, the indirect reflected light is collected by the collecting lens and detected by the detector, which is a reverse-oriented photodiode without a direct view of the objects, as shown in Fig. 1. The fusion, imaging and encryption of the multiple objects are achieved using the intensities detected by the detector. The recovered results with different compression ratios are shown in Fig. 2. The SNRs of the recovered images are lower than those in other cases because the light collected by the detector was weaker because of the low reflectivity of white A4 paper. Although scattering reduces the SNR, the four objects shown in Fig. 2 can still be perceived. The ability to image multiple objects through scattering media makes our system valuable in numerous applications, ranging from optical communication through turbulent atmosphere to microscopic imaging in turbid tissues.


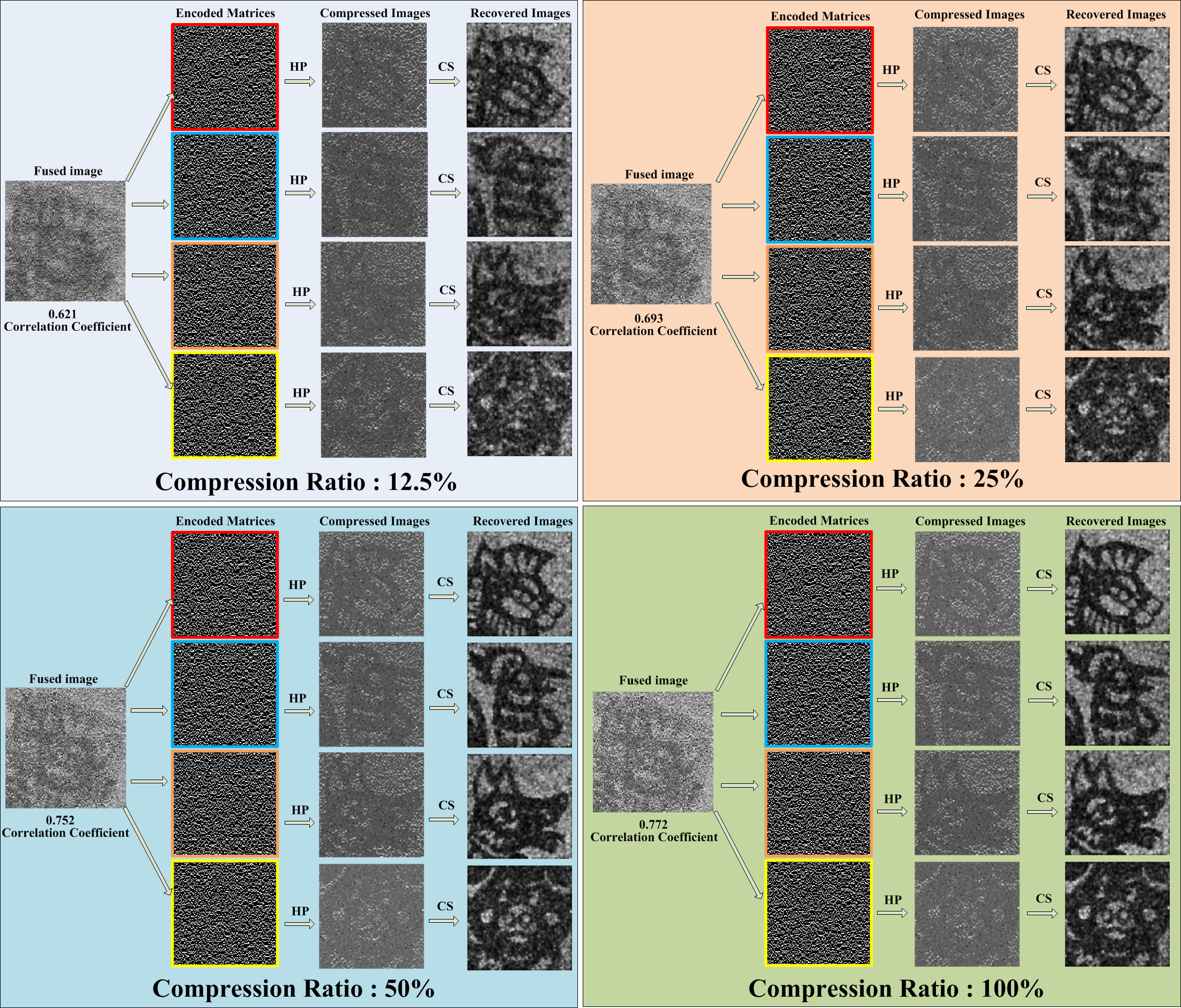


Fig. 2. Multiple objects reconstructed with different compression ratios using indirect reflected light in the third case. Scene reconstructed with different compression ratios according to the correlation coefficient between the recovered images and the reconstruction utilizing a complete Hadamard basis (100% compression ratio).

We imitate a brute force attack. Fifty encoded matrices with a certain level of accuracy and different distributions are generated as decryption keys. In each attack, the reconstruction using encoded matrices with a certain level of accuracy retrieve the information. The minimum and maximum values of the correlation coefficients in fifty different cases are shown in Fig. 3. For the scattering environment, because of the lower SNR of the fused image, the quality of recovered images shown in Fig. 3 deteriorates rapidly relative to the previous situation. These findings are confirmed by the correlation coefficients shown in the figure. Note that when the compression ratio exceeds a certain value, the qualities of the recovered images are not necessarily enhanced as the compression ratio increases. According to the results, the accurate encryption information sought by the eavesdropper becomes more difficult to obtain in a scattering environment than in a clear medium. Thus, a scattering medium can improve the encryption performance.


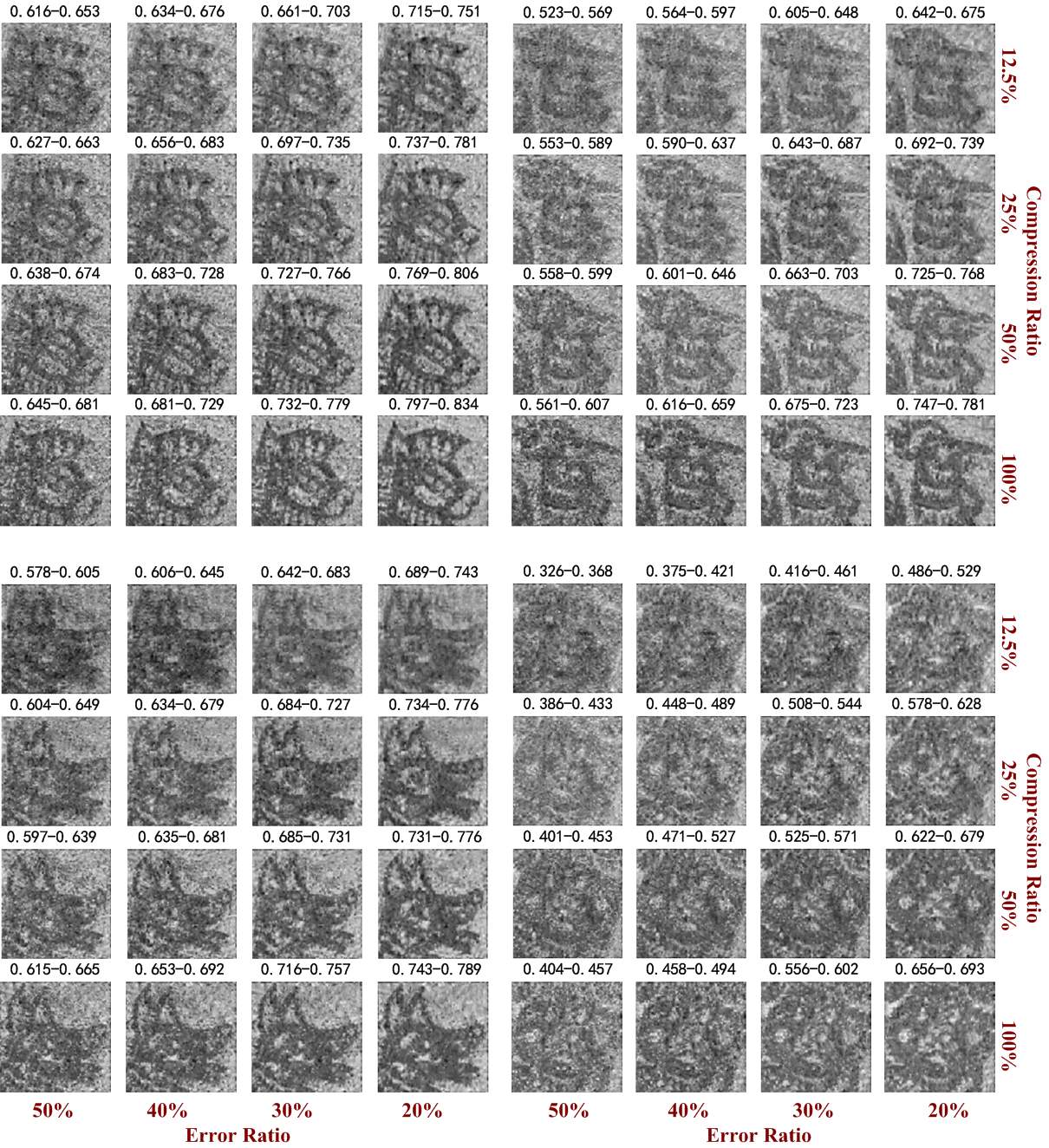


Fig. 3. Image encryption experiment using indirect reflected light in the third case. The results obtained under error ratios of 50%, 40%, 30% and 20% for the encoded matrices are shown. The numbers indicate the correlation coefficients between the recovered images and the reconstruction utilizing the complete Hadamard basis for direct reflected light (100% compression ratio).

**Image comparison based on correlation coefficients**

To compare image acquisitions, we used the correlation coefficients between the under-sampled images and a reference image. This coefficient ranges from zero to one, depending on the resemblance of both images. Our reference image is always the image acquired without under-sampling (i.e., measuring at the Nyquist-Shannon criterion). The correlation coefficient is calculated with the following function:

$r=\frac{\sum_{m} \sum_{n} (A_{mn}-\bar{A})\circ(B_{mn}-\bar{B})}{\sqrt{\sum_{m} \sum_{n} (A_{mn}-\bar{A})^{2}\circ\sum_{m} \sum_{n} (B_{mn}-\bar{B})^{2}}}$ , (12)

where A and B are the image matrices with indices *m* and *n*, respectively, and $\bar{A}$ and $\bar{B}$ represent the mean values of the elements in A and B.
